# Historical Isolation *versus* Recent Long-Distance Connections between Europe and Africa in Bifid Toadflaxes (*Linaria* sect. *Versicolores*)

**DOI:** 10.1371/journal.pone.0022234

**Published:** 2011-07-14

**Authors:** Mario Fernández-Mazuecos, Pablo Vargas

**Affiliations:** Real Jardín Botánico, CSIC, Madrid, Spain; J. Craig Venter Institute, United States of America

## Abstract

**Background:**

Due to its complex, dynamic and well-known paleogeography, the Mediterranean region provides an ideal framework to study the colonization history of plant lineages. The genus *Linaria* has its diversity centre in the Mediterranean region, both in Europe and Africa. The last land connection between both continental plates occurred during the Messinian Salinity Crisis, in the late Miocene (5.96 to 5.33 Ma).

**Methodology/Principal Findings:**

We analyzed the colonization history of *Linaria* sect. *Versicolores* (bifid toadflaxes), which includes c. 22 species distributed across the Mediterranean, including Europe and Africa. Two cpDNA regions (*rpl*32-*trn*L^UAG^ and *trn*K-*mat*K) were sequenced from 66 samples of *Linaria*. We conducted phylogenetic, dating, biogeographic and phylogeographic analyses to reconstruct colonization patterns in space and time. Four major clades were found: two of them exclusively contain Iberian samples, while the other two include northern African samples together with some European samples. The bifid toadflaxes have been split in African and European clades since the late Miocene, and most lineage and speciation differentiation occurred during the Pliocene and Quaternary. We have strongly inferred four events of post-Messinian colonization following long-distance dispersal from northern Africa to the Iberian Peninsula, Sicily and Greece.

**Conclusions/Significance:**

The current distribution of *Linaria* sect. *Versicolores* lineages is explained by both ancient isolation between African and European populations and recent events of long-distance dispersal over sea barriers. This result provides new evidence for the biogeographic complexity of the Mediterranean region.

## Introduction

Studying the role of biogeographic barriers as limiting factors for plant range expansion and gene flow allows investigation of the causes behind population differentiation and speciation (e. g. [1], [2]). A remarkable spatial and temporal complexity makes the Mediterranean basin an ideal geographic framework for this approach. The abundance of islands, peninsulas, straits and mountains, and the complex history of climate and sea-level changes have created changing opportunities for plant dispersal and colonization across different barriers [3], [4]. The Messinian Salinity Crisis (MSC), in the late Miocene (5.96 to 5.33 Ma) [5] has long been considered the last major window of opportunity for plant colonization across the Mediterranean [6]. Desiccation of the Mediterranean Sea during this age formed land bridges that facilitated plant range expansion, including colonization events between Africa and Europe (e. g. [7]). After the opening of the Strait of Gibraltar and the refilling of the Mediterranean basin (Miocene-Pliocene boundary, 5.33 Ma), isolation on both continental plates may have led to vicariant processes between European and African lineages. This intercontinental isolation made long-distance seed dispersal essential for range expansion over the newly created marine barriers.

Several factors may account for the differences in ability to expand a range over biogeographic barriers. Multiple patterns of colonization found in the Mediterranean suggest that habitat specificity, rather than morphological traits for dispersal, may have been crucial limiting factors [8], [9], [10], [11]. Certainly, recurrent seed colonization over sea barriers, specifically the Strait of Gibraltar, has been shown to be more likely if favourable ecological conditions are widespread, regardless of whether plants possess special mechanisms for long-distance dispersal [9], [11]. In other cases, however, long-term isolation between Iberian and NW African populations appears to have occurred, again irrespectively of seed dispersal mechanisms [7], [12], [13], [14]. Although the role of the Strait of Gibraltar as a biogeographic bridge or barrier has been assessed in several studies (see [8] for a revision), little is still known about the impact of the Mediterranean Sea as a large barrier for floristic exchange between Europe and Africa in the last 6 Ma [9], [15], [16]. Time-calibrated phylogenetic and phylogeographic analyses of Mediterranean plant lineages are required to understand dispersal, colonization and isolation processes across the changing sea barriers of the Mediterranean basin [9], [11].

Toadflaxes (*Linaria* Mill.) constitute the largest genus within the tribe Antirrhineae. It comprises nearly 150 species classified into seven sections (see Table S1), and it has been suggested to be monophyletic by previous phylogenetic results [17]. The genus has its diversity centre in the Mediterranean region, where all seven sections and c. 70% of species are present. Five sections are distributed both in the European and African parts of the Mediterranean region [18]. Small seeds of *Linaria* are enclosed in capsules, and may or may not be surrounded by an encircling wing. Therefore, this group constitutes a good system to analyze intercontinental colonization processes at the species and population levels, as well as the role of sea barriers in isolation.

The plastid genome (cpDNA) has been widely used in plant phylogenetics and phylogeography given its haploid and non-recombinant nature. When it is also maternally inherited, as commonly in angiosperms, including *Linaria* [19], cpDNA lineages can be used to infer patterns of colonization by seeds [20]. Recently developed methods, such as relaxed molecular-clock dating [21] and model-based biogeographic reconstruction [22], [23] allow estimating absolute dating of biogeographic events. Here, we applied a multi-scale approach based on the analysis of cpDNA sequences in order to reconstruct the colonization history of *Linaria* sect. *Versicolores* over marine barriers of the Mediterranean basin.

## Methods

### Study taxa

Section *Versicolores* (Bentham) Wettst. represents one of the most distinctive subdivisions of *Linaria* (toadflaxes), due to the bifid style with discrete stigmatic areas (bifid toadflaxes), a trait not found in the rest of the genus [18], [24], [25]. Seeds are wingless, and thus show no obvious capability for long-distance dispersal. Table 1 summarizes the infrasectional taxonomy followed in this paper, primarily based on [18] (but see also [26], [27]). The group comprises 22 species primarily of lowland habitats and is mainly distributed in the western Mediterranean, on both sides of the Mediterranean Sea (Europe/Africa). According to the most recent taxonomic revision [18], [24], [25], [26], [27], sect. *Versicolores* includes eight European endemics, nine northern African endemics, one species from northern Africa and the Middle East (*L. tenuis*), and four species co-occurring in southern Europe and northern Africa: *L. incarnata*, *L. pedunculata* and *L. gharbensis* are found on both sides of the Strait of Gibraltar, while *L. multicaulis* is distributed in Morocco, Algeria, Tunisia, Sicily and Calabria. *Linaria hellenica*, an eastern Mediterranean species narrowly distributed in Greece, has been included within the African *L. tenuis* by some authors, on the basis of morphological characters [28]. The taxonomic complexity of sect. *Versicolores* has long been recognized, particularly the poorly understood African taxa [18]. Therefore, species delimitation based on extant taxonomy must be taken with caution. Phylogenetic studies are needed for systematic delimitation of specific and subspecific taxa.

**Table 1:** Samples of the studied taxa and populations of *Linaria* sect.

Taxon	Distribution	Number of sampled populations	Sequence/haplotype codes
*Linaria* sect. *Versicolores* (Bentham) Wettst.			
Subsect. *Versicolores*			
*L. algarviana* Chav.	SW Portugal (Algarve)	1	Ib6
*L. bipartita* (Vent.) Willd.	W Morocco	2	17, 17
*L. bordiana* Santa & Simonneau	NW Africa	2	13, 14
*L. clementei* Haenseler ex Boiss.	S Spain (Málaga)	2	Ib2, Ib1
*L. gharbensis* Batt. & Pitard	NW Africa, SW Spain	4	5, 6, 7, 16
*L. hellenica* Turrill	S Greece	1	4
*L. imzica* Gómiz	S Morocco (Anti Atlas)	1	21
*L. incarnata* (Vent.) Sprengel	SW Iberian Peninsula, NW Morocco	6	Ib6, Ib6, Ib7, 7, 8, 23
*L. maroccana* Hooker fil.	Morocco (mainly High Atlas)	2	22, 23
*L. multicaulis* (L.) Miller			
subsp. *multicaulis*	Sicily, S Italy (Calabria)	1	12
subsp. *aurasiaca* (Pomel) D.A. Sutton	Tunisia, NE Algeria	1	11
subsp. *galioides* (Ball) D.A. Sutton	Morocco (High Atlas)	2	2, 3
subsp. *heterophylla* (Desf.) D.A. Sutton	NW Africa	5	1, 16, 18, 19, 20
*L. pedunculata* (L.) Chaz.	S Iberian Peninsula, NW Africa, Balearic islands	9	9, 9, 9, 9, 9, 9, 9, 9, 9
*L. pinifolia* (Poiret) Thell.	Tunisia, Algeria	1	16
*L. pseudoviscosa* Murb.	Tunisia	1	10
*L. salzmannii* Boiss.	S Spain (Málaga)	1	Ib1
*L. spartea* (L.) Chaz.	Iberian Peninsula, S France	2	Ib3, Ib6
*L. tenuis* (Viv.) Sprengel	N Africa, Middle East	2	11, 11
*L. tingitana* Boiss. & Reuter	NW Africa	2	15, 16
*L. viscosa* (L.) Chaz.			
subsp. *viscosa*	S Iberian Peninsula	2	Ib4, Ib5
subsp. *spicata* (Coutinho) D.A. Sutton	SE Iberian Peninsula	2	Ib1, Ib1
*L. weilleri* Emberger & Maire	S Morocco (Anti Atlas)	1	21
Subsect. Elegantes (Viano) D.A. Sutton			
*L. elegans* Cav.	NW Iberian Peninsula	2	Le1, Le2
*L. nigricans* Lange	SE Spain (Almería)	2	Ln1, Ln2

*Versicolores*, including geographic distribution and *rpl*32-*trn*L^UAG^/*trn*K-*mat*K sequence/haplotype codes (as in Figs. 2, 3, 5 and 6).

### Sampling strategy and DNA sequencing

We sampled a total of 57 populations of *Linaria* sect. *Versicolores* (one individual per population), including representatives of 25 species and subspecies (Table 1, Table S1). We failed to sample *L. dissita*. This is a poorly known African taxon of minor relevance for our objectives, because it seems to be closely related to other African species, with which it could even be con-specific [18]. We made special emphasis in the sampling of multiple populations of morphologically variable, widely distributed, and intercontinental species in order to test biogeographic hypotheses. To test the monophyly of section *Versicolores*, we also sampled nine additional species representing the remaining six sections of *Linaria*. One species of *Chaenorhinum* and one of *Antirrhinum* were included as the outgroup on the basis of a previous phylogeny of the tribe Antirrhineae [17]. Plant material was collected in the field and dried in silica gel or obtained from herbarium collections (RNG, MA, ATH, UPOS, SALA; Table S1).

Total genomic DNA was extracted using DNeasy Plant Mini Kit (QIAGEN Inc., California). A pilot study using 6 samples of different species was performed to find the most variable sequences among 14 plastid DNA regions previously used in phylogenetic and phylogeographic analyses [29], [30]. DNA regions were amplified in an Eppendorf Mastercycler Epgradient S (Westbury, NY) or a MJ Research PTC-200 (Massachusetts) thermal cycler. After 1 min pretreatment at 95°C, PCR conditions were: 30 cycles of 1 min at 94°C, 1–2 min at 48–55°C and 1–2 min at 72°C. In certain reactions, a volume of 1 µL of bovine serum albumine (BSA) at 1 mg ml^−1^ was included in each 25 mL reaction to improve the efficiency of amplification. Amplified products were treated with ExoSAP-IT (USB Corporation, Ohio) and submitted to Macrogen Inc. (Seoul, South Korea) for sequencing. Resulting sequence data were assembled and edited using Geneious Pro v5 [31]. We identified two highly variable cpDNA regions: *rpl*32-*trn*L^UAG^ [29] and *trn*K-*mat*K [32] and then the sequencing of these regions was extended to every sampled individual. In order to facilitate amplification and sequencing from partially degraded DNA obtained from herbarium specimens, we designed the following internal primers for both regions and used them in combination with the standard primers: rpl32-trnL_intF (5′-CATTTCCAAGGTGGGGAGTCT-3′), rpl32-trnL_intR (5′-AGAAATAGGTTGATGGGGA-3′), trnK-matK_intF1 (5′-ACCTGTCTCCGAGGTATCTA-3′), trnK-matK_intF2 (5′-GGGGTTTGCATTTATTGTGG-3′), trnK-matK_intR1 (5′-CACGATCATGAGCAAACGCA-3′), and trnK-matK_intR2 (5′-CCACAATAAATGCAAACCCC-3′). We also designed a reverse primer specific to *Linaria* sect. *Versicolores* for *trn*K-*mat*K: 1470R_Lvers (5′- AAGATGTTGATCGTAAATCC-3′). All sequences were submitted to GenBank (see Table S1 for accession numbers).

### Phylogenetic analysis

Sequences of each cpDNA region (*rpl*32-*trn*L^UAG^ and *trn*K-*mat*K) were aligned using MAFFT 6 [33] with default parameters, and further adjustments were made by visual inspection. The two regions were combined in a single matrix, and phylogenetic relationships were assessed using maximum parsimony (MP), maximum likelihood (ML) and Bayesian inference (BI). The MP analysis was performed in TNT 1.1 [34] using a heuristic search with 10,000 replicates saving two most-parsimonious trees per replicate, followed by a second heuristic search retaining all best trees and using the trees obtained in the previous 10,000 replicates as the starting ones. Bootstrap support (MP-BS) of clades was assessed using 1000 standard replicates. For ML and BI analyses, the simplest model of sequence evolution that best fits the sequence data (GTR for *trn*K-*mat*K and GTR+G for *rpl*32-*trn*L^UAG^ and the combined dataset) was determined under the Akaike Information Criterion (AIC) in jModelTest 0.1.1 [35]. ML was implemented in PhyML 3.0 [36] with 500 non-parametric bootstrap replicates (ML-BS). BI was performed in MrBayes v3.1.2 [37] using two searches with 10 million generations each and a sample frequency of 1000. The two regions were partitioned and unlinked. Chain convergence was assessed with Tracer 1.4 [38], and a 50% majority rule consensus tree with Bayesian posterior probabilities (PP) of clades was calculated to obtain the Bayesian estimate of phylogeny after removing the first 10% generations as burn-in.

### Estimation of divergence times

To estimate divergence times among *Linaria* sect. *Versicolores* lineages, we implemented a relaxed molecular-clock approach in BEAST v.1.6.0 [21], [39], a software that simultaneously estimates tree topology and node ages. Identical sequences of the *rpl*32-*trn*L^UAG^/*trn*K-*mat*K matrix were removed from the analysis. Gaps were treated as missing data. Since no reliable fossils of *Linaria* are known to date, only molecular estimates were available for temporal calibration of the tree. The divergence time between *Chaenorhinum* and *Linaria* was modelled as a normal distribution with mean = 29 Ma and standard deviation = 4.6, on the basis of an estimate obtained in a relaxed molecular-clock analysis of tribe Antirrhineae (P. Vargas *et al.*, unpublished). This analysis incorporates a calibration of 97 Ma for the crown-age of Lamiales [40] and minimum stem-age constraints for Lamiales families and tribes based on five fossils: *Fraxinus wilcoxiana* (Oleaceae, Middle Eocene) [41], *Catalpa rugosa* (Bignoniaceae, Early-Middle Oligocene) [42], *Ajuginucula smithii* (Lamiaceae, Early-Middle Oligocene) [42], *Gratiola tertiaria* (Gratioleae, Miocene) [43] and *Plantaginacearumpollis* (Plantaginaceae s.str., Middle Miocene) [44]. All these fossils have been considered reliable and proposed as calibration points for molecular dating in previous studies [45], [46], [47]. The substitution rate variation was modelled using an uncorrelated lognormal distribution, and a Birth-Death process [48] was employed as tree prior. Two MCMC analyses were run for 10 million generations, with a sample frequency of 1000. Both chains were combined using LogCombiner 1.4.8, after discarding the first 10% of sampled generations as burn-in. Parameter analysis in Tracer 1.4 [38] confirmed adequate sample size, with ESS values above 650 and plots showing equilibrium. Trees were summarized in a maximum clade credibility (MCC) tree obtained in TreeAnotator 1.4.8 and visualized in FigTree 1.1.2.

### Biogeographic reconstruction

In order to infer colonization events of *Linaria* sect. *Versicolores* across the Mediterranean, biogeographic reconstructions were conducted delimiting four areas based on the distribution of sampled taxa and the presence of marine barriers: northern Africa (A); Iberian Peninsula (I); Sicily (S); and Greece (G). We employed a model-based maximum-likelihood approach for ancestral area optimization: the dispersal-extinction-cladogenesis (DEC) model implemented in Lagrange 2.0.1 [22]. This analysis requires a fully dichotomous tree, and thus the BEAST output is appropriate. Given the polyphyly of several species in the phylogenetic analysis of the full dataset (see below), we did not attempt a biogeographic reconstruction using the complete phylogeny and species distribution ranges. Instead, we employed the phylogeny (MCC tree) obtained in the BEAST analysis. Outgroup taxa were pruned, and distribution ranges of plants containing the same sequence, instead of species ranges, were attached to tree tips. Although the inclusion of an outgroup has been recommended for DEC analysis [49], we did not proceed because poor sample of *Linaria* as a whole impeded finding a reliable sister group to sect. *Versicolores*.

Lagrange uses DEC modelling to compute the likelihood of range inheritance scenarios at nodes in a phylogeny, and allows incorporation of information about changing dispersal opportunities associated to geological events (e. g. area connections). We compared four models (M0–M3) differing in maximum number of areas allowed in ancestral ranges and constancy of dispersal rates through time. In M0, the maximum number of areas was unconstrained and dispersal rate was constant through time. M1 incorporated a maximum of two areas in ancestral ranges, based on current distributions. In M2, dispersal rate was set to vary according to historical connections among areas: it was maximum (λ_D_ = 1) during the MSC (5.96–5.33 Ma), when the contact between the Eurasian and African plates and the desiccation of the Mediterranean Sea eliminated marine barriers among areas; conversely, dispersal rate was set to a lower value (λ_D_ = 0.1) during the time intervals before and after the Messinian event, when marine barriers were active. Finally, model M3 combined constraints on maximum number of areas and dispersal rates from M1 and M2. To asses the statistical significance of likelihood differences among models, we employed the conventional cut-off value of two log-likelihood units [50].

Reconstructions described above rely on the single MCC tree. To account for uncertainty in tree topology and node ages, possibly affecting the reconstruction at the root node, we repeated the analyses under the four DEC models over a sample of 100 trees from the posterior distribution of the BEAST analysis. Then we summarized the resulting scenarios of range inheritance at the root node obtained in the 100 analyses under each DEC model.

For comparison with a parsimony-based reconstruction method, we also performed dispersal-vicariance analyses (DIVA) [51]. To account for phylogenetic uncertainty and uncertainty in area optimization, DIVA analyses were implemented in S-DIVA [52], a software that statistically evaluates the alternative ancestral ranges at nodes based on a set of trees [53], [54]. As input, we used the complete tree distribution obtained from BEAST and the final MCC tree. We conducted two analyses allowing for a maximum of two or four areas in ancestral ranges and with the option “Allow reconstruction” in effect, which calculates the probabilities of ancestral ranges at nodes following [54]. The two analyses were repeated unchecking the mentioned option, thus applying the method of [53].

### Haplotype data analysis

We analyzed the colonization history of intercontinental lineages through a haplotype network approach. Genealogical relationships among haplotypes of clades III and IV (see below) were inferred using the statistical parsimony algorithm [55], as implemented in TCS 1.21 [56]. The maximum number of differences resulting from single substitutions among haplotypes was calculated with 95% confidence limits, treating gaps as missing data.

## Results

### Phylogenetic relationships

**Figure 1:**
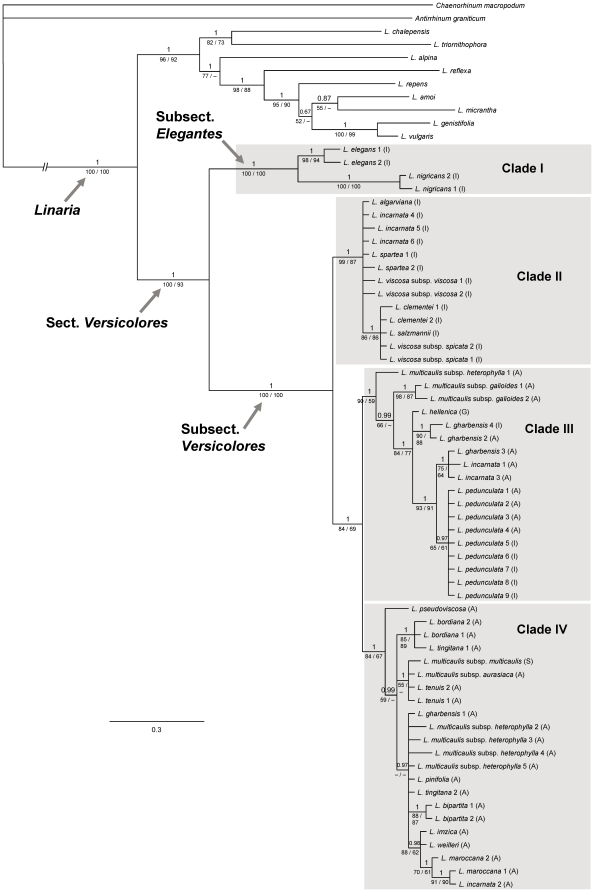
Plastid phylogeny of bifid toadflaxes. Phylogenetic relationships of 57 samples representing 25 species and subspecies of *Linaria* sect. *Versicolores* based on the combined analysis of cpDNA regions *rpl*32-*trnL* ^UAG^ and *trn*K-*mat*K. The majority-rule consensus tree obtained in the Bayesian analysis is shown. Numbers above branches are Bayesian posterior probabilities. Numbers below branches are maximum likelihood/maximum parsimony bootstrap values. A hyphen (–) indicates no bootstrap support over 50%. Populations of the same species are numbered as in Table S1. Geographic location of sect. *Versicolores* samples is shown in brackets. I – Iberian Peninsula; A – northern Africa; S – Sicily; G – Greece.

Two of the 14 cpDNA variable regions tested (*rpl*32-*trn*L^UAG^ and *trn*K-*mat*K) rendered the highest number of reliable nucleotide substitutions. The characteristics of the two sequenced cpDNA regions sequenced are summarized in Table 2. The total aligned length of the combined dataset was 2066 bp, and 187 of the 395 variable sites were parsimony-informative. The 50% majority-rule consensus tree of the Bayesian analysis is shown in Fig. 1. The ML tree showed the same topology, while the strict consensus tree of the MP analysis was fully congruent, although with a lower resolution and support values (Fig. 1).

**Table 2:** Characteristics of the *rpl*32-*trn*L^UAG^ and *trn*K-*mat*K sequences obtained for *Linaria* sect.

	*rpl*32-*trn*L^UAG^	*trn*K-*mat*K
Aligned length (bp)	830	1236
Ungapped length range	568–754	1209–1227
Pairwise % identity	94.7	98.3
Variable characters	209	186
Parsimony-informative characters	105	82
Mean % G+C content	22.2	32.3

*Versicolores* samples and the outgroup.

All three phylogenetic analyses recognized section *Versicolores* as monophyletic with high support values. Within the section, two well-supported sister clades were retrieved, which support the two morphology-based subsections: *Elegantes*, formed by the two sister species *L. elegans* and *L. nigricans* (clade I; PP = 1; ML-BS = 100%; MP-BS = 100%); and *Versicolores*, encompassing the remaining species (PP = 1; ML-BS = 100%; MP-BS = 100%). Two major lineages were found within the latter subsection. The first one (clade II; PP = 1; ML-BS = 99%; MP-BS = 87%) contained Iberian samples, including all the Iberian endemics, *L. spartea* and Iberian accessions of *L. incarnata*, and was sister to a second lineage formed by clades III and IV (PP = 1; ML-BS = 84%; MP-BS = 69%). These two clades primarily contained northern African samples (including those of *L. incarnata*), together with samples from Sicily and Greece, and Iberian samples of *L. gharbensis* and *L. pedunculata*. Accessions of the same species or subspecies were retrieved as monophyletic groups only for *L. elegans*, *L. nigricans*, *L. multicaulis* subsp. *galioides*, *L. pedunculata* and *L. bipartita*, while polyphyly was clearly retrieved for *L. incarnata*, *L. gharbensis*, *L. multicaulis* subsp. *heterophylla* and *L. tingitana*.

### Divergence times

**Figure 2:**
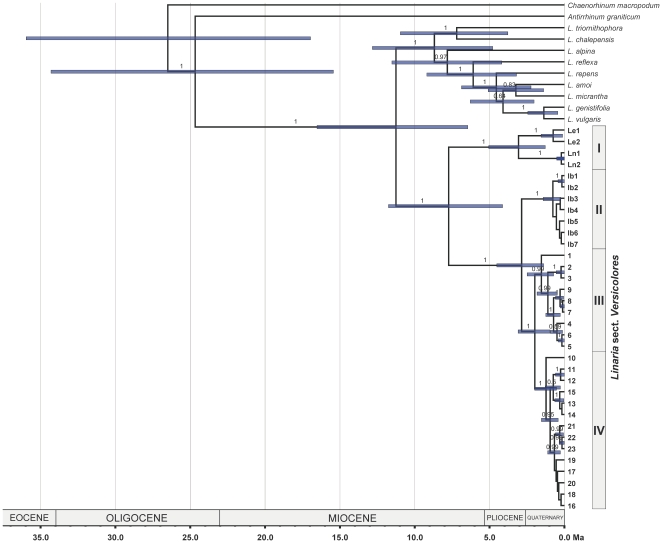
Molecular dating analysis. Maximum clade credibility tree produced by relaxed molecular-clock analysis of *rpl*32-*trnL* ^UAG^ and *trn*K-*mat*K sequences in BEAST, excluding sequence identities of *Linaria* sect. *Versicolores* and the outgroup. Node bars represent the 95% highest posterior density intervals for the divergence time estimates of clades with posterior probabilities above 0.50. Values above branches indicate Bayesian posterior probabilities. Major clades of the study group are indicated.

Values of standard deviation of the uncorrelated lognormal relaxed clock (0.25) and coefficient of variation (0.24) for rate heterogeneity within our cpDNA dataset indicated a low deviation from a strict molecular clock. The topology of the MCC tree (Fig. 2) was congruent with that of the other phylogenetic analyses. The chronogram supported a crown-age for *Linaria* sect. *Versicolores* around the late Miocene, while a Pliocene or early Quaternary divergence for the two main lineages of subsect. *Versicolores* (Fig. 2; Table 3). Lineage differentiation within clades II, III and IV appears to have occurred during the Quaternary. A very recent divergence (<1 Ma) was supported for European accessions (sequences 4, 6, 9 and 12) within the mainly northern African clades (III, IV).

**Table 3:** Divergence dates of major plastid clades of *Linaria* sect.

Clade	Crown age (Ma)	95% HPD interval
Sect. *Versicolores*	7.73/7.60	4.13–11.75
Subsect. *Elegantes* (clade I)	3.06/2.94	1.28–5.05
Subsect. *Versicolores* (clade II+III+IV)	2.86/2.77	1.39–4.51
Clade II	0.77/0.71	0.24–1.42
Clade III+IV	1.97/1.91	0.96–3.09
Clade III	1.54/1.49	0.72–2.49
Clade IV	1.23/1.19	0.54–1.98

*Versicolores*, presented as mean/median crown ages plus 95% highest posterior density (HPD) intervals based on relaxed molecular-clock analysis of *rpl*32-*trn*L^UAG^ and *trn*K-*mat*K sequences in BEAST.

### Biogeographic reconstruction

**Figure 3:**
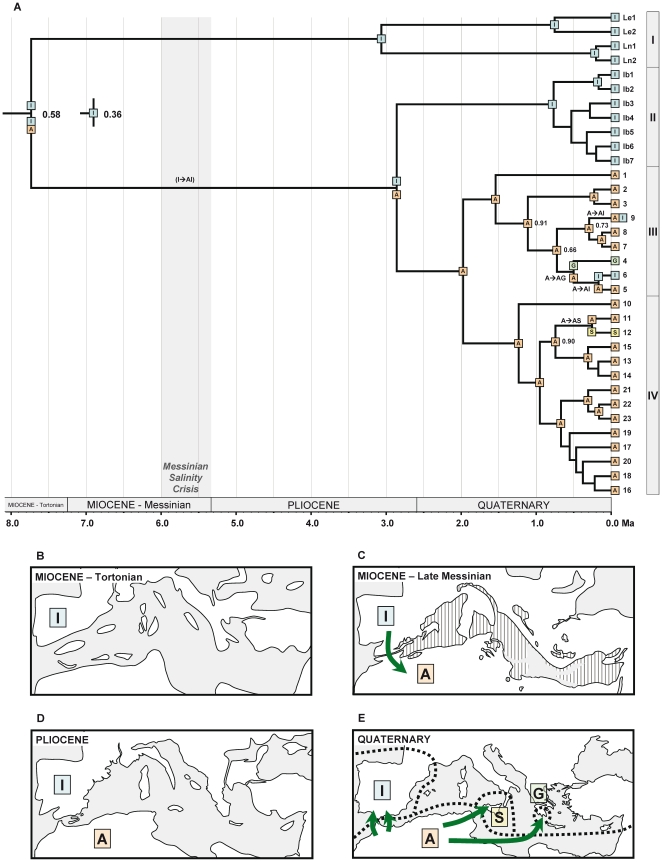
Hypothesis of colonization history based on DEC analysis. (A) Biogeographic reconstruction based on dispersal-extinction-cladogenesis modelling implemented in Lagrange using the single MCC tree from the BEAST analysis (Fig. 2) after pruning outgroup taxa. Coloured squares represent maximum-likelihood range inheritance scenarios reconstructed under model M3 for nodes with PP above 0.5. Ranges inherited from widespread ancestors following cladogenesis are shown at the base of diverging branches, while single-area ancestral ranges are shown at nodes. Inferred events of dispersal along branches are also illustrated. When a node has alternative scenarios within 2 log-likelihood units of the optimal reconstruction, the relative probability (fraction of the global likelihood) for the optimal reconstruction is indicated. Given the relevance of the root node for the early colonization history of the group, the two alternative reconstructions are displayed, and the dispersal event inferred under the second best scenario (relative probability 0.36) is shown in brackets along the branch leading to subsect. *Versicolores*. (B–D) Hypothesis of colonization history of *Linaria* sect. *Versicolores* across the Mediterranean basin since the Late Miocene, based on phylogenetic, dating and biogeographic reconstruction results, as well as geological information. Coloured squares represent the range occupied by the group during each period, and arrows indicate hypothetical colonization events. Paleogeographic maps are based upon [77] (white – emerged areas; grey – submerged areas; lined – desiccating areas). Areas delimited for reconstructions are displayed in E. I – Iberian Peninsula; A – northern Africa; S – Sicily; G – Greece.

The four biogeographic models tested in Lagrange (Table 4) produced results with similar likelihood values. Model M2 received the highest log-likelihood (−28.28), but all other models fell within 2 log-likelihood units of the optimal one, and thus M2 was not significantly supported as the best model. The four models produced slightly different reconstructions of colonization history, with models M0 and M2 (both with unconstrained maximum number of areas in ancestral ranges) resulting in a higher number of alternative scenarios falling within 2 log-likelihood units of the optimal reconstruction. For example, seven alternative scenarios were obtained for the most recent common ancestor of sequences 4–9 in models M0 and M2, while three and four scenarios were obtained for models M1 and M3 respectively, even though all four models inferred an African ancestor (A|A, being the area on the left the one inherited by the upper daughter branch in Fig. 3, and the area on the right the one inherited by the lower daughter branch) as the optimal reconstruction. For simplicity, we show the optimal reconstruction under the most biologically realistic model (M3, higher dispersal rate during the MSC and a maximum of two ancestral areas at nodes) in Fig. 3A. In fact, the four models inferred the same optimal reconstruction in all but two nodes: the root node, as discussed below, and the most recent common ancestor of sequences 4–6. In the latter, G|AI was the optimal scenario in models M0 and M2, while G|A was for models M1 and M3, thus placing a dispersal event to the Iberian Peninsula along different branches.

**Table 4:** Results for the biogeographic models tested in Lagrange, including values of log-likelihood (lnL), dispersal rate (λ_D_), extinction rate (λ_E_) and maximum-likelihood scenarios of range inheritance for the tree root and selected clades.

	M0	M1	M2	M3
lnL	−28.57	−28.75	−28.28	−28.65
λ_D_	0.038	0.046	0.425	0.493
λ_E_	0.000	0.000	0.000	0.000
Sect. *Versicolores* (root)	I (0.50), I|AI (0.37)	I|AI (0.46), I (0.46)	I|AI (0.51), I (0.39)	I|AI (0.58), I (0.36)
Subsect. *Elegantes* (clade I)	I	I	I	I
Subsect. *Versicolores* (clade II+III+IV)	I|A (0.80), I|AI (0.07)	I|A	I|A (0.82), I|AI (0.06)	I|A
Clade II	I	I	I	I
Clade III+IV	A (0.79), AI|A (0.10)	A	A (0.81), AI|A (0.10)	A
Clade III	A (0.79), A|AI (0.09)	A	A (0.81), A|AI (0.10)	A
Clade IV	A	A	A	A

When a bar separates two ranges, the first range is inherited by the upper daughter branch in Fig. 3 and the second range is inherited by the lower daughter brach. If a node has multiple scenarios within 2 log-likelihood units of the optimal reconstruction, the two most likely scenarios are shown, and the relative probability of each is indicated in brackets. I – Iberian Peninsula; A – northern Africa.

The optimal reconstruction under model M3 (Fig. 3A) supports a common ancestor of sect. *Versicolores* distributed both in the Iberian Peninsula and northern Africa (relative probability 0.58). An Iberian-only range was inherited by one of its daughter lineages, leading to the common ancestor of subsect. *Elegantes* (clade I), while a widespread range (IA) was inherited by the daughter lineage leading to subsect. *Versicolores* (clades II–IV). In the cladogenetic event at the base of subsect. *Versicolores*, this widespread ancestor yielded two daughter lineages inheriting mutually exclusive ranges: Iberian Peninsula for the ancestor of clade II and northern Africa for the ancestor of clades III–IV. Subsequent dispersal events from northern Africa to the Iberian Peninsula and Greece in clade III, and to Sicily in clade IV gave rise to current ranges of sublineages and species in these lineages.

**Figure 4:**
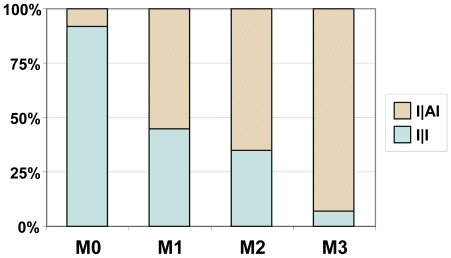
Uncertainty of area reconstruction at the root. A sample of 100 trees from the posterior distribution of the BEAST analysis was analyzed in Lagrange under the four DEC models. Bars summarize the proportion of trees yielding a certain maximum-likelihood scenario for the root node. Only two possible scenarios were retrieved: I|AI and I|I.

Under the second best scenario at the root node (relative probability 0.36), an Iberian common ancestor of sect. *Versicolores* produced two Iberian daughter lineages, one of which dispersed to northern Africa, giving rise to a widespread western Mediterranean ancestor of subsect. *Versicolores* (Fig. 3A). The uncertainty on the range of the common ancestor of sect. *Versicolores* was maintained under the other DEC models, with an Iberian ancestor supported by model M0 and a widespread ancestor by models M1 and M2 (Table 4). When taking into account the uncertainty on topology and branch lengths by analyzing 100 trees from the posterior distribution of the BEAST analysis (Fig. 4), we obtained contrasting results under different DEC models. An Iberian ancestor was supported under model M0 as the maximum-likelihood scenario for a high percentage of trees (92%). On the contrary, a widespread western Mediterranean ancestor was supported under model M3 for a similar percentage of trees (93%). Models M1 and M2 yielded a higher uncertainty (see Fig. 4).

**Figure 5:**
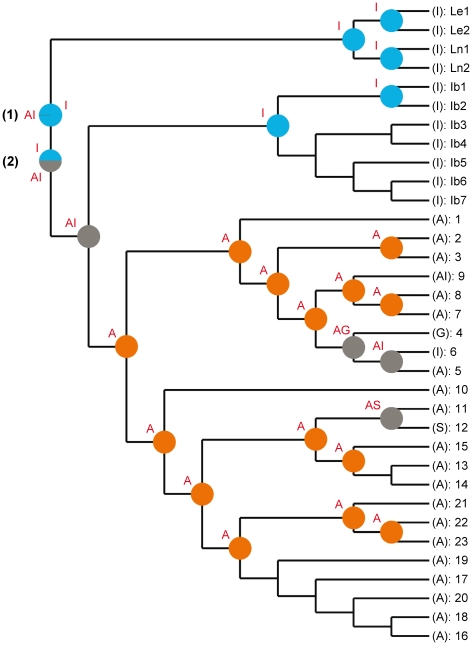
Statistical DIVA analysis. Biogeographic reconstruction based on statistical dispersal-vicariance analysis as implemented in S-DIVA, with the maximum number or areas at ancestral nodes set to two. The tree is the same as in Fig. 2 after pruning outgroup taxa. Pie charts at nodes represent marginal probabilities for ancestral areas. Different ways of calculating probabilities of ancestral ranges did not affect the result, except for the root node, where reconstructions following [54] (1) and [53] (2) are shown. I – Iberian Peninsula; A – northern Africa; S – Sicily; G – Greece.

Results of S-DIVA analyses were mostly congruent with DEC inferences. A higher resolution was also found when the maximum number of areas was set to two (Fig. 5). As in DEC reconstructions, there was uncertainty on the range of the root node (I or AI). An Iberian ancestor was strongly supported under calculations of ancestral probabilities following [54]. However, an Iberian and a widespread western Mediterranean ancestor were equally supported under the method of [53]. In all other nodes, the same ancestral ranges were obtained in the four S-DIVA analyses (results not shown), which fit the scenarios inferred by DEC modelling as shown in Fig. 3A.

### Haplotype network

**Figure 6:**
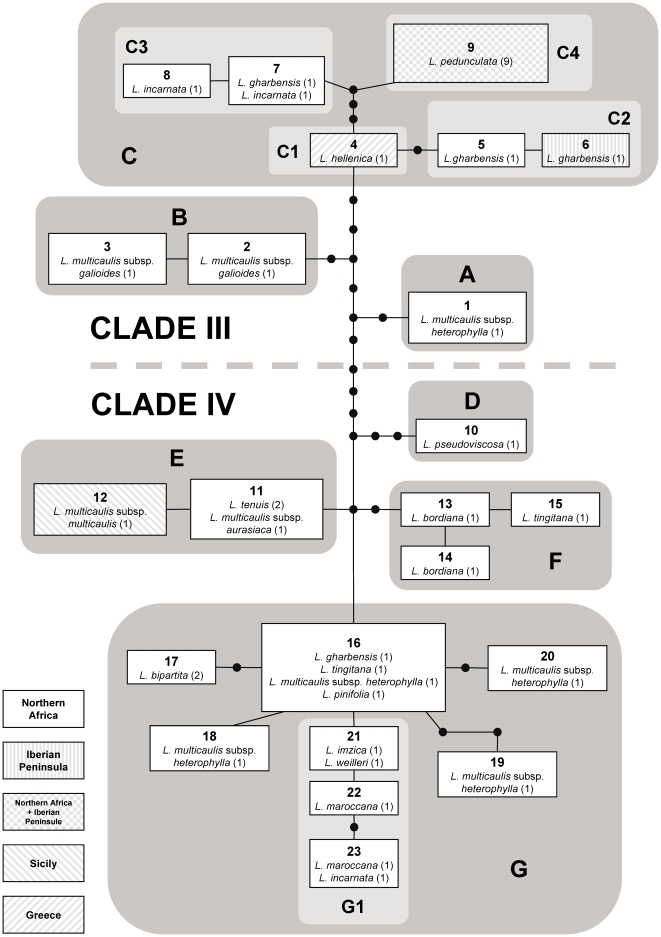
Haplotype network of primarily northern African clades. Statistical parsimony network of cpDNA haplotypes (indicated as numbered squares) of clades III and IV. Lines represent single nucleotide substitutions; dots indicate absent haplotypes (extinct or not found). Taxa harbouring each haplotype are shown within the squares, with the number of sequenced individuals indicated in brackets. Geographic distribution of haplotypes is shown, and main clades and lineages mentioned in the text are delimited.

**Figure 7:**
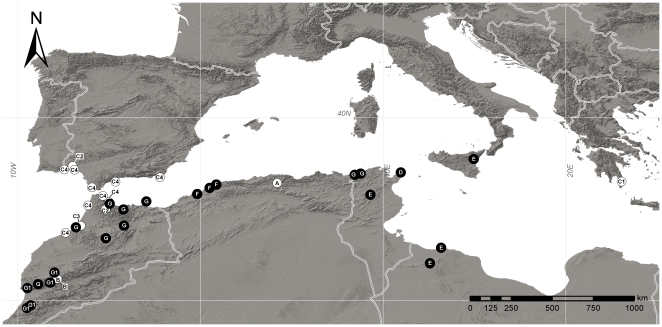
Geographic distribution of cpDNA haplotype lineages of clades III and IV. Lineages are named as in Fig. 6. Samples of clade III are shown with white circles, while samples of clade IV are shown with black circles.

The haplotype network analysis of the primarily African lineages (clades III, IV) (Fig. 6) distinguished seven main haplotype lineages (A–G) with a high number (16) of internal missing haplotypes separating them. Haplotypes depicted in Fig. 6 correspond to distinct sequences of dating and biogeographic analyses (Figs. 2, 3 and 5). The geographic distribution of haplotype lineages and sublineages is illustrated in Fig. 7. Only two of these lineages (C and E) were distributed on both Europe and northern Africa. Lineage E included accessions of *L. tenuis* and *L. multicaulis* subsp. *aurasiaca* from central northern Africa together with the accession of *L. multicaulis* subsp. *multicaulis* from Sicily (tip haplotype 12). Lineage C contained similar numbers of European and African samples. Interestingly, a Balkan-African-Iberian connection was obtained because the internal haplotype (4) in lineage C was found in the Greek *L. hellenica*, and was connected to a Moroccan sample of *L. gharbensis* (Haplotype 5), and then to the tip haplotype (6) of the Iberian accession of *L. gharbensis* (see sublineage C2 in Figs. 6 and 7). All nine samples of *L. pedunculata* (five Iberian and four northern African) yielded the same widely-distributed tip haplotype (9, sublineage C4 in Figs. 6 and 7).

A lack of agreement between haplotype lineages and species delimitation in northern Africa is apparent for some taxa, specially the widely-distributed and morphologically variable *L. multicaulis*. Samples of different subspecies of *L. multicaulis* are included in four separate haplotype lineages, whereas one of its haplotypes (16) is shared by three other species and has a wide distribution from Morocco to Tunisia. By contrast, taxa endemic to narrower areas (including subspecies of *L. multicaulis*) frequently belong to equally narrowly distributed haplotype lineages: B (High Atlas; *L. multicaulis* subsp. *galioides*), E (central northern Africa and Sicily; *L. multicaulis* subsp. *multicaulis*, *L. multicaulis* subsp. *aurasiaca*), F (NW Algeria; *L. bordiana*); and G1 (Atlas and Anti-Atlas mountains; *L. maroccana*, *L. imzica*, *L. weilleri*). The most remarkable incongruence between taxonomy, geography and phylogenetic results was also observed in the phylogenetic analyses (Fig. 1).

## Discussion

### Complex taxonomy and reliability of biogeographic reconstruction

In our biogeographic analyses, we explicitly designed a morphological and geographic sample to overcome methodological issues associated with the complex taxonomy of *Linaria*. Biogeographic reconstructions are often performed on the basis of a gene tree (or a tree resulting from concatenation of several DNA regions) including a single individual to represent the entire distribution of each species. This is appropriate when the major biogeographic patterns of a large lineage are the focus [57], [58], [59], provided that populations of each species constitute a natural group. When inferring colonization events at a finer scale, as in our case, this approach is more problematic. Assuming naturalness of species and equalling the obtained tree to the species tree may lead to spurious reconstructions under a scenario of rapid, convergent or paralell evolution. Indeed, the prolific diversification of subsect. *Versicolores*, possibly accompanied of incomplete sorting of ancestral polymorphisms and hybridization, accounts for the frequent non-monophyly of con-specific samples of our phylogeny [60] (see Fig. 1). In addition, species delimitation is particularly difficult in *Linaria* due to the poor knowledge of some taxa [18], [24], [25]. Under this complex scenario, we consider our cpDNA phylogeny and lineage-based area reconstruction the most appropriate approach to circumvent methodological problems to infer colonization patterns. For instance, this approach has revealed that *L. incarnata* might be a polyphyletic taxon. Iberian and Moroccan populations are currently recognized as the same species [18], [27], [61], [62], yet some morphological differences have been suggested [18]. Our analyses separate them in primarily Iberian or African clades (II and III–IV), and the similar morphologies are more easily explained by parallel evolution than by intercontinental dispersal. Likewise, there is uncertainty on the taxonomic status of Greek populations named as *L. hellenica* by some authors [18], [63] or assigned to *L. tenuis* by others [28]. Our analyses clearly place *L. hellenica* and African samples of *L. tenuis* in separate lineages. In the case of the polyphyletic *L. gharbensis*, Iberian populations were formerly described as *L. heterophylla* subsp. *tartessiana* [64] or *L. tartessiana* [65], but have only been assigned to *L. gharbensis* in the last treatments [27], [66]. In this case, a close phylogeographic relationship between the Iberian and one of the African samples of *L. gharbensis* is obtained in our analyses (lineage C2 in Fig. 6), but the species is nevertheless retrieved as polyphyletic.

### Miocene origin of the bifid toadflaxes

Given that section *Versicolores* is strongly supported as a monophyletic group by our phylogenetic analysis, the origin and colonization of its lineages can be reliably inferred. In addition, naturalness of sect. *Versicolores* is supported by a diagnostic morphological synapomorphy (discrete stigmatic areas), which is unique in a genus of over 150 species, as already stated by [18]. The first dichotomy within the group agrees with the infrasectional taxonomy established in [18], with subsections *Elegantes* and *Versicolores* being well supported as monophyletic sister groups.

The crown-age of sect. *Versicolores* based on our dating analysis supports an origin of the group around the Middle to Late Miocene. The same might be true for several major lineages of *Linaria* used as the outgroup, as shown by the chronogram in Fig. 2. Increasing of aridity in the Mediterranean area during the Cenozoic culminated in the Messinian Salinity Crisis [6] and the establishment of the Mediterranean climate [67], and these climatic changes had a major impact in the western Mediterranean flora [68]. Assuming an herbaceous (annual), xeromorphic ancestor (as most species of sect. *Versicolores*, including those of the basal-most lineages), we interpret specialization of bifid toadflaxes to xeric environments to be associated with climate changes of the Miocene. As species diversification in sect. *Versicolores* (primarily affecting subsect. *Versicolores*) mostly happened during the Pliocene and Quaternary, specialization to annuality and other xerophytic characteristics (waxes covering the whole plant, linear leaves) may have been retained in the bifid toadflaxes since the Miocene.

### Historical Europe-Africa isolation

Four major lineages of sect. *Versicolores* (I, II, III, IV) were found in our phylogenetic analysis, which are primarily distributed in the Iberian Peninsula or northern Africa. Our biogeographic reconstructions are not conclusive on the ancestral area of sect. *Versicolores*, except for the statistical DIVA analysis performed following [54], which clearly supports an Iberian ancestor. Regardless of the uncertainty, we are favouring the hypothesis of an Iberian ancestor (Fig. 3B) over a widespread (Iberian-African) ancestor considering the fact that both DEC and DIVA have a tendency to infer widespread ancestors [49], [50], [69]. If this is the case, a dispersal event from the Iberian Peninsula to northern Africa would have occurred along the branch leading to subsect. *Versicolores*, which is congruent with a range expansion during the Messinian connection between Europe and Africa (Fig. 3C). Alternative hypotheses are plausible, and a better-supported inference for the ancestral area of sect. *Versicolores* will probably be obtained when the sister group to sect. *Versicolores* is determined.

Despite uncertainty at the root, it is clearly inferred that the Mediterranean Sea acted as an effective sea barrier for colonization by the wingless seeds of *Linaria* sect. *Versicolores* after the refilling of the basin at the Miocene-Pliocene boundary (5.33 Ma). Subsect. *Elegantes* (lineage I) inherited an Iberian ancestral range from the common ancestor, and subsequently gave rise to the two extant Iberian species (*L. elegans* and *L. nigricans*). A widespread Iberian-northern African ancestor of subsect. *Versicolores* was inferred. The well-supported subdivision of its range between Iberian (clade II) and northern African (clades III and IV) sister groups is congruent with lineage isolation that followed the opening of the Strait of Gibraltar in the early Pliocene (Fig. 3D). Geographic isolation between African and European populations and taxa has previously been described [14], [70], [71], [72], [73], [74]. In *Linaria* sect. *Versicolores*, the possibility remains that Pliocene colonizations between Africa and Europe occurred, but the resulting genetic footprint was erased. New connections between extant lineages of both areas have occurred only in recent times, as discussed below.

### Recent colonizations over the Mediterranean Sea

The disjunct distribution of any taxa in two areas currently separated by a dispersal barrier may be explained by two alternative scenarios: colonization before barrier formation or dispersal after barrier formation. Distinguishing between these two hypotheses constitutes one of the most discussed topics in historical biogeography [51], [75], [76]. The widely-used dispersal-vicariance analysis [51] does not *a priori* take into account the relative timing of lineage divergence and barrier formation, and therefore is not ideally suited for this task [49]. Instead, a combination of time-calibrated phylogenetic data with geologic and paleogeographic information can provide robust inferences, and this is the approach taken by modern analytical methods [22], [50] and employed in our study.

Indeed, the well-known paleogeographic framework of the Mediterranean basin (e. g. [77] allows for successful implementation of this approach to analyze the biogeographic history of taxa in this area [9], [11], [78], [79], [80], [81]. Species level (phylogenetic) and population level (phylogeographic) analyses have tested disjunct distributions in southern Europe and northern Africa [70], [82], [83], [84]. However, only a few studies are placed in a time-calibrated framework and are thus able to date colonization events in relation to the last land connection between both continents during the Messinian. For example, colonization along a Messinian land bridge between Europe and Africa has been proposed for the xerophytic species *Androcymbium gramineum* [7] and the *Campanula broussonetiana*/*C. transtagana* lineage [12]. On the other hand, post-Messinian long-distance colonization over the Mediterranean Sea has been strongly supported in several species of *Cistus*, a typically Mediterranean genus [9], [11]. Like *Cistus*, *Linaria* sect. *Versicolores* diversified mainly after the establishment of the Mediterranean climate and underwent events of post-Messinian intercontinental colonization despite lacking any special mechanism for long-distance dispersal. At least four colonization events from Africa to Europe in clades III and IV are supported by our biogeographic reconstructions (Fig. 3 and 5), and the relaxed molecular-clock analysis (Fig. 2) unambiguously places all these events in the last one million years (Quaternary, Fig. 3E). The recent expansion of open Mediterranean habitats may have favoured intercontinental colonization after long-distance dispersal in recent times [8].

Intercontinental colonization may have been more likely in those regions where African and European land masses have been closer, particularly the straits of Gibraltar and Sicily. Over the Strait of Gibraltar area, at least two events of jump dispersal are inferred (lineages C2 and C4 of clade III). Recent studies indicate that the Strait of Gibraltar may not have constituted such an important barrier for plant colonization as previously thought (reviewed in [8]). Several events of recent colonization between Africa and Europe in this area have been documented within each of four *Cistus* species [9], [11] and also in *Linaria* sect. *Supinae* (J.L. Blanco Pastor & P. Vargas, unpublished). In our case, in lineage C2, the restricted populations of *L. gharbensis* in SW Spain [27] are closely related to populations of the same species in northern Morocco, while in lineage C4, all nine samples of *L. pedunculata* (five Iberian and four northern African) shared a common and exclusive cpDNA haplotype (Figs. 6 and 7). This result indicates a recent and rapid expansion of *L. pedunculata* across maritime dunes and sandy beaches of Southern Iberia and NW Africa, which may have been facilitated by the autocompatibility of this species (M. Fernández-Mazuecos & P. Vargas, unpublished). Marine dispersal may also have played a role, as was the case for other coastal species of the Mediterranean area [85].

Over the Strait of Sicily, our results support a recent colonization by lineage E (clade IV): the tip haplotype 12, found in Sicily (*L. multicaulis* subsp. *multicaulis*), is closely related to haplotype 11, found in three samples from Tunisia and Libya (*L. multicaulis* subsp. *aurasiaca* and *L. tenuis*) (Figs. 6 and 7). Similarly to the Strait of Gibraltar, the Strait of Sicily was formed with the refilling of the Mediterranean at the beginning of the Pliocene, which flooded the Messinian land bridge that last connected northern Africa and the landmass that later became Sicily (Figs. 3C and 3D) [86], [87]. Although the Strait is now 140 km wide, it may have been reduced to c. 50 km during Pleistocene glaciations, which does not affect our hypothesis of jump dispersal from northern Africa to Sicily across a marine sea barrier. Long-distance dispersal over the Strait of Sicily has also been documented for the *Anthemis secundiramea* group [16], [88] and for *Cistus salviifolius* [9].

The fourth inferred event of Africa-Europe colonization is harder to interpret. It is shown by the presence of haplotype 4 in Greek populations of the endangered *L. hellenica* [18], [63]. Our analyses placed this haplotype in a lineage mostly including samples from the area surrounding the Strait of Gibraltar (lineage C; Figs. 6 and 7), and very unconnected to the morphologically and geographically related samples of *L. tenuis*. Although our data clearly support long-distance dispersal from northern Africa, a deeper sampling, particularly in NE Africa, is needed to confirm whether the Greek populations are indeed the result of colonization from the Strait of Gibraltar area. In any case, long-distance colonization between the western and eastern Mediterranean has been suggested for other taxa, such as the coastal *Calystegia soldanella* [89], and again the widely-distributed Mediterranean shrub *Cistus salviifolius* [9].

In summary, the causes behind the disjunct distribution of bifid toadflaxes in the Iberian Peninsula and northern Africa have been carefully addressed in a time-calibrated phylogenetic framework. The Mediterranean Sea acted as a relatively effective barrier for lineage connections of *Linaria* sect. *Versicolores* since the end of the Miocene. In fact, Iberian and northern African lineages appear to have diversified in isolation after the Pliocene refilling of the basin. However, some colonization events from northern Africa to Europe in very recent times (<1 Ma) are clearly attributable to intercontinental colonization, despite the absence of specific mechanisms for long-distance dispersal. The small size of the seeds and the abundance of open and sandy habitats in the Mediterranean region probably favoured these events. Therefore, processes of both geographic isolation and long-distance dispersal may have taken place and explain the current distribution of *Linaria* sect. *Versicolores* lineages across the Mediterranean basin.

## Supporting Information

Table S1 **Voucher specimens and GenBank accession numbers of sampled taxa and populations of Linaria sect.** *Versicolores* and the outgroup. Sequence/haplotype codes are shown for ingroup samples (as in Figs. 2, 3, 5 and 6).
(PDF)
