# Supplementary material for: Historical Isolation versus Recent Long-Distance Connections between Europe and Africa in Bifid Toadflaxes (Linaria sect. Versicolores)
Source: PLoS One. 2011 Jul 14;6(7):e22234. doi: 10.1371/journal.pone.0022234 (PMC3136523; doi:10.1371/journal.pone.0022234)
Supplement: Table S1 — Voucher specimens and GenBank accession numbers of sampled taxa and populations of Linaria sect. Versicolores and the outgroup. Sequence/haplotype codes are shown for ingroup samples (as in Figs. 2, 3, 5 and 6). (PDF) [file pone.0022234.s001.pdf]

Taxon	(Population No.) Sampled locality	Voucher	Sequence / haplotype code	*rpl*32-*trn*L^{UAG} accession no.	*trn*K-*mat*K accession no.
Antirrhinum L.					
Antirrhinum graniticum Rothm.	Spain, Cáceres, Trujillo	P. Vargas 213PV06 (MA)	-	JF694120	JF694188
Chaenorhinum (DC.) Reichenb.					
Chaenorhinum macropodum (Boiss. & Reuter) Lange	Spain, Málaga, Cómpeta	P. Vargas 27PV08 (MA)	-	JF694119	JF694187
Linaria Mill.					
Linaria sect. *Diffusae* (Bentham) Wettst.					
L. reflexa (L.) Chaz.	Algeria, Algiers	J.J. Aldasoro A9799 (MA)	-	JF694129	JF694197
Linaria sect. *Linaria*					
L. vulgaris Miller	France, Chamonix	B. Estébanez s.n. (MA)	-	JF694126	JF694194
Linaria sect. *Macrocentrum* D.A. Sutton					
L. chalepensis (L.) Miller	Cyprus, Larnaca, Cape Kiti	Iter Mediterraneum IV 294 (MA)	-	JF694127	JF694195
Linaria sect. *Pelisserianae* Valdés					
L. triornithophora (L.) Willd.	Spain, Cáceres, Puerto de Perales	M. Fernández-Mazuecos 18MF07 (MA)	-	JF694128	JF694196
Linaria sect. *Speciosae* (Bentham) Wettst.					
L. genistifolia (L.) Miller	Turkey, Hadim-Bezkir	J.J. Aldasoro & M.L. Alarcón A9751 (MA)	-	JF694124	JF694192
L. repens (L.) Miller	Spain, Cuencia, Beteta	M. Fernández-Mazuecos 54MF09 (MA)	-	JF694125	JF694193
Linaria sect. *Supinae* (Bentham) Wettst.					
L. alpina (L.) Miller	Spain, Huesca, Bujaruelo	J. Güemes s.n. (MA)	-	JF694121	JF694189
L. amoi Campo ex Amo	Spain, Málaga, Cómpeta	M. Fernández-Mazuecos, A.D. Forrest & P. Vargas 30PV08 (MA)	-	JF694122	JF694190
L. micrantha (Cav.) Hoffmanns. & Link	Spain, Alicante, Vall de Gallinera	J.X. Soler & M. Signes 1530-JXS (MA)	-	JF694123	JF694191
Linaria sect. *Versicolores* (Bentham) Wettst.					
Subsect. *Versicolores*					
L. algarviana Chav.	Portugal, Cabo de São Vicente	M. Fernández-Mazuecos 11MF09 (MA)	Ib6	JF694130	JF694198
L. bipartita (Vent.) Willd.	(1) Morocco, Rabat	S.L. Jury with R.G. Wilson 18558 (RNG)	17	JF694131	JF694199
	(2) Morocco, Tamri	S.L. Jury, B. Tahmi & T.M. Upson 14293 (RNG)	17	JF694132	JF694200
L. bordiana Santa & Simonneau	(1) Algeria, Mostaganem – Tenes	Davis 51833 (RNG)	13	JF694134	JF694202
	(2) Algeria, Sidi Lakhdar	D.A. & S.J. Sutton 172 (RNG)	14	JF694133	JF694201
L. clementei Haenseler ex Boiss.	(1) Spain, Málaga, Alhaurín de la Torre	M. Fernández-Mazuecos, A.D. Forrest & P. Vargas 7MF08 (MA)	Ib2	JF694135	JF694203
	(2) Spain, Málaga, Coín	M. Fernández-Mazuecos & J. Ramírez 24MF09 (MA)	Ib1	JF694136	JF694204
L. gharbensis Batt. & Pitard	(1) Morocco, Bou Ahmed	S.L. Jury, A. Taleb, T.M. Upson & G.S Waltens 13406 (RNG)	16	JF694137	JF694205
	(2) Morocco, Chefchaouen	J. Montserrat & J. Vicens JMM-4193/5 (RNG)	5	JF694177	JF694245
	(3) Morocco, Rabat	S.L. Jury & R.G. Wilson 18559 (RNG)	7	JF694138	JF694206
	(4) Spain, Huelva, Gibraleón	M. Fernández-Mazuecos, J.L. Blanco & E. Sánchez-Gullón 7MF09 (MA)	6	JF694139	JF694207
L. hellenica Turrill	Greece, Kambos	Unknown collector (ATH)	4	JF694140	JF694208
L. imzica Gómiz	Morocco, Jbel Imzi	F. Gómiz s.n. (MA)	21	JF694141	JF694209
L. incarnata (Vent.) Sprengel	(1) Morocco, Kenitra-Khemisset	S. Martín-Bravo, I. Pulgar, F.J. Fernández, G.C. Mazo 34SMB06 (MA)	8	JF694142	JF694210
	(2) Morocco, Marrakech	S.L. Jury 14151 (RNG)	23	JF694143	JF694211
	(3) Morocco, Salé	J. Lambinon & G. van den Sande nº95/Ma/333 (RNG)	7	JF694144	JF694212
	(4) Spain, Badajoz, Alburquerque	M. Fernández-Mazuecos 9MF09 (MA)	Ib6	JF694145	JF694213
	(5) Spain, Salamanca, Pelabravo	M. Fernández-Mazuecos & P. Vargas 39MF09 (MA)	Ib7	JF694146	JF694214
	(6) Spain, Huelva, Valverde del Camino (=*L. onubensis* Pau)	V. Valcarcel & P. Vargas 5PV08 (MA)	Ib6	JF694147	JF694215
L. maroccana Hooker fil.	(1) Morocco, Imouzzer Valley	M. Ait Lafkih s.n. (RNG)	23	JF694148	JF694216
	(2) Morocco, Marrakech – Tizi-n-Test	S.L. Jury, B. Tahiri & T.M. Upson 14209 (RNG)	22	JF694149	JF694217
L. multicaulis (L.) Miller					
subsp. *multicaulis*	Italy, Sicily, Etna	I. Álvarez *et al.* IA1622 (MA)	12	JF694150	JF694218
subsp. *aurasiaca* (Pomel) D.A. Sutton	Tunisia, El Kesra	Davis & Lamond 57154 (RNG)	11	JF694151	JF694219
subsp. *galioides* (Ball) D.A. Sutton	(1) Morocco, Oukaimedem	P. Jiménez Mejías, E. Narbona, A.J. Chaparro & M. Parra 200PJM05 (UPOS)	2	JF694152	JF694220
	(2) Morocco, Yagour	A. Kool with H.J. Boer, P. Domínguez 904 (RNG)	3	JF694153	JF694221
subsp. *heterophylla* (Desf.) D.A. Sutton	(1) Algeria, Tikjda	Davis 53078 (RNG)	1	JF694154	JF694222
	(2) Morocco, Azrou	M. Fernández-Mazuecos & J.C. Moreno 15MF08 (MA)	20	JF694155	JF694223
	(3) Morocco, Beni-Hadifa	B. Guzmán 108bBGA04 (MA)	18	JF694156	JF694224
	(4) Morocco, Jbel Tazekka	E. Rico, S. Andrés & M. Santos SA-221 (SALA)	19	JF694157	JF694225
	(5) Tunisia, Tabarka	J.J. Aldasoro A2888 (MA)	16	JF694158	JF694226
L. pedunculata (L.) Chaz.	(1) Morocco, Chefchaouen	M.A. Mateos, M.C. Reina, G. Sangalli, N. Sardón & B. Valdés s.n. (RNG)	9	JF694163	JF694231
	(2) Morocco, Larache	Silvestre, G.-Rowe & Vilches s.n. (RNG)	9	JF694167	JF694235
	(3) Morocco, Mdiq	W. Lippert 24229 (RNG)	9	JF694164	JF694232
	(4) Morocco, Mohammedia	Aparicio, Rowe & Silvestre s.n. (RNG)	9	JF694165	JF694233
	(5) Portugal, Monte Gordo	M. Fernández-Mazuecos & J.L. Blanco 61MF10 (MA)	9	JF694166	JF694234
	(6) Spain, Almería, Cabo de Gata	M. Fernández-Mazuecos 30MF09 (MA)	9	JF694159	JF694227

	(7) Spain. Cádiz, Barbate	E. Sánchez-Gullón s.n. (MA)	9	JF694160	JF694228
	(8) Spain, Huelva, Marismas del Odiel	M. Fernández-Mazuecos, J.L. Blanco & E. Sánchez-Gullón 4MF09 (MA)	9	JF694161	JF694229
	(9) Spain, Málaga, Mijas	M. Fernández-Mazuecos & J. Ramírez 27MF09 (MA)	9	JF694162	JF694230
L. pinifolia (Poiret) Thell.	Tunisia, El Kala	A. Dubuis, H. Maurel & R. Rhamoun s.n. (RNG)	16	JF694168	JF694236
L. pseudoviscosa Murb.	Tunisia, El Haouaria	P. Wilkin & E.J. Wellens 231 (RNG)	10	JF694169	JF694237
L. salzmannii Boiss.	Spain, Málaga, El Chorro	M. Fernández-Mazuecos & J. Ramírez 19MF09 (MA)	Ib1	JF694170	JF694238
L. spartea (L.) Chaz.	(1) Spain, Madrid, Colmenar	P. Vargas 101PV07 (MA)	Ib6	JF694171	JF694239
	(2) Spain, Soria, Tardelcuende	M. Fernández-Mazuecos, A. Quiroga, S.C. Herrera & D. Orgaz 14MF07 (MA)	Ib3	JF694172	JF694240
L. tenuis (Viv.) Sprengel	(1) Libya, Tripoli	Davis & Boulos 50581 (RNG)	11	JF694174	JF694242
	(2) Libya, Gebel Nefoussa	Davis 49632 (RNG)	11	JF694173	JF694241
L. tingitana Boiss. & Reuter	(1) Algeria, El Macta	D.A. & S.J. Sutton 383 (RNG)	15	JF694175	JF694243
	(2) Morocco, Cap des Trois Fourches	T.M. Upson and Ait Lafkih, M. Hassan, G.S. Walters 14012 (RNG)	16	JF694176	JF694244
L. viscosa (L.) Chaz.					
subsp. *viscosa*	(1) Spain, Huelva, Marismas del Odiel	M. Fernández-Mazuecos, J.L. Blanco & E. Sánchez-Gullón 6MF09 (MA)	Ib4	JF694178	JF694246
	(2) Spain, Huelva, Matalascañas	M. Fernández-Mazuecos & J.L. Blanco 1MF09 (MA)	Ib5	JF694179	JF694247
subsp. *spicata* (Coutinho) D.A. Sutton	(1) Spain, Jaén, Cazorla	M. Fernández-Mazuecos 49MF09 (MA)	Ib1	JF694181	JF694249
	(2) Spain, Málaga, Cómpeta	M. Fernández-Mazuecos, A.D. Forrest & P. Vargas 9MF08 (MA)	Ib1	JF694180	JF694248
L. weilleri Emberger & Maire	Morocco, Tirhmi	Miller, Russell & Sutton s.n. (RNG)	21	JF694182	JF694250
Subsect. Elegantes (Viano) D.A. Sutton					
L. elegans Cav.	(1) Spain, Orense, San Xoán de Río	M. Fernández-Mazuecos 45MF08 (MA)	Le2	JF694184	JF694252
	(2) Spain, Ávila, Plataforma de Gredos	E. Amat s.n. (MA)	Le1	JF694183	JF694251
L. nigricans Lange	(1) Spain, Almería, Tabernas	P. Vargas 3PV08 (MA)	Ln2	JF694186	JF694254
	(2) Spain, Almería, Cabo de Gata	M. Fernández-Mazuecos 29MF09 (MA)	Ln1	JF694185	JF694253
